# Randomized Controlled Trial: Does the Use of Occlusive Hydrocolloid Silver-Containing Wound Dressing after Sternotomy Reduce Surgical Site Infection after Cardiac Surgery?

**DOI:** 10.3390/life14091061

**Published:** 2024-08-24

**Authors:** Ryan Chaban, Kathrin Dohle, Ahmed Ghazy, Martin Oberhoffer, Christian-Friedrich Vahl, Hendrik Treede, Mehmet Oezkur

**Affiliations:** Department of Cardiovascular Surgery, University Hospital of Johannes Gutenberg University Mainz, 55131 Mainz, Germany; ryan.chaban@unimedizin-mainz.de (R.C.); ahmed.ghazy@unimedizin-mainz.de (A.G.); martin.oberhoffer@unimedizin-mainz.de (M.O.); hendrik.treede@unimedizin-mainz.de (H.T.); mehmet.oezkur@unimedizin-mainz.de (M.O.)

**Keywords:** surgical site infection, cardiac surgery, occlusive hydrocolloid silver-containing wound dressing, advanced dressings, postoperative care, postoperative measures, single-center randomized controlled trial

## Abstract

(1) Background: To reduce the incidence of surgical site infections (SSIs) following median sternotomy in cardiac surgery, we compared an occlusive hydrocolloid silver-containing wound dressing (OHSCWD) with a standard wound dressing. (2) Methods: This study was designed as a single-center randomized controlled trial. The primary endpoint was the overall rate of incidence of any kind of SSI. Secondary endpoints were the number of dressing changes, the severity of SSIs, and whether there was a need for treatment. Wounds were monitored daily until the seventh and on the 30th postoperative day. (3) Results: Of the 423 patients included, 352 were analyzed. No differences in demographics, cardiovascular risk factors, intraoperative processes, and postoperative care were found between both groups. Additionally, the incidence or extent of SSI showed no significant differences between the two groups. (4) Conclusions: In summary, out of all pre-, intra-, and postoperative factors, the contribution of postoperative wound care to the development of SSIs appears to play a subordinate role. However, by offering equivalent wound protection and a reduced number of dressing changes, OHSCWD after median sternotomy in cardiac surgery patients could be a good alternative to standard dressings from the point of view of the patient, the staff, and the clinic.

## 1. Introduction

Surgical site infections (SSIs) are among the most common complications of cardiac surgery [1,2]. The incidence varies in the recent literature but is between 5–15% for superficial infection and 1–5% for deep infection [3,4,5,6,7,8,9,10,11]. 

Preventing SSIs is crucial because of their serious consequences: patients with deep sternal infections have an increased mortality risk of up to 31% [12,13]. Patients not only suffer from significantly longer hospitalization periods of up to 6 weeks but are handicapped in their mobility and quality of life [14]. Furthermore, the costs for treating patients increase with the occurrence of SSIs [12,15,16].

Risk factors for the development of an SSI are well-researched and include those that cannot be influenced by the clinical team, such as advanced age, genetic predisposition, obesity, malnutrition, diabetes, nicotine and alcohol abuse, comorbidity, and immunosuppressive or hormonal treatment [4,17]. However, there are also factors that are directly related to the surgery, such as wound size, intraoperative exposure of the surgical site to microbes, operation time, tissue trauma, tissue blood perfusion, closure technique, blood transfusion, low-output syndrome, intensive care treatment, postoperative hygiene, and wound care technique [18].

All current guidelines and recent reviews [19,20,21,22,23,24] suggest, as preventive measures in the field of cardiac surgery, nasal and skin decolonization, hair removal outside of the operating room, the use of skin preparation with a remanence effect, and perioperative glycemic control.

As our clinic already follows these recommendations, we focused on optimizing postoperative wound care in order to further reduce our incidence of SSI. Therefore, we chose an occlusive hydrocolloid silver-containing wound dressing (OHSCWD), which is an established method for preventing and treating wound infections in other surgical fields [25,26,27]. Dressings with a hydrocolloid adhesive layer and a polyurethane cover should provide adequate wound protection during the first seven days when the body’s own wound barrier is still developing. Silver ions are highly reactive, binding to tissue proteins and causing structural changes in the bacterial cell wall, as well as in the intracellular and nuclear membranes, ultimately leading to cellular distortion and loss of viability. Additionally, silver ions further exhibit their bacteriostatic properties by binding to and denaturing bacterial DNA and RNA, thereby inhibiting bacterial replication [28]. They can interfere with thiol groups and provoke the generation of reactive oxygen species. These mechanisms appear to be essential factors for its antibacterial efficacy [29] and thus for the reduction of microbacterial colonization [30,31]. 

Due to the increasing development of bacterial resistance caused by the disproportionate use of antibiotics, among other reasons, there is an urgent need to establish alternative antimicrobial agents for wound treatment. Continuously applicable systems such as delayed-release dressings can improve therapeutic efficacy, reduce side effects, and increase local bioavailability. In contrast to silver nanoparticles, antimicrobial nanoparticles that release an antibiotic have the disadvantage of a variable release process, which can impair the efficacy of the drug. There are also other nanoparticles that can cause hypothermia in some patients by triggering a photothermal effect [32]. The distribution and accumulation of non-biodegradable nanoparticles in organs (mainly the liver and spleen) can potentially lead to toxicity for the host [33]. Accordingly, nanosilver-based dressings are repeatedly the focus of discussion in humans due to the toxicity of silver ions. However, toxicity can be controlled by controlling the size of the nanoparticles, the storage conditions, and the concentration of silver ions [34]. The dressing we used in this study releases a concentration of silver ions that only act locally and are barely absorbed systemically, which is why the risk of significant toxicity from the silver ions of this dressing with consequent permanent organ damage appears to be negligible [35]. However, local allergic reactions are occasionally reported.

Whether OHCSWD can be used in a preventive manner in patients undergoing coronary artery bypass grafting (CABG) still remains unclear. Therefore, the aim of this study is to investigate if OHSCWD prevents SSI in patients undergoing CABG surgery.

## 2. Materials and Methods

This study was designed as a single-center randomized controlled trial and screened 762 consecutive patients undergoing CABG surgery in our department from January 2018 to July 2019. We included 423 patients, of which 352 were analyzed.

This study was conducted in accordance with the relevant guidelines for good clinical and good scientific practice defined by the 1964 Helsinki Declaration and after obtaining clearance from the Ethics Board of Rhineland-Palatinate, Germany (application Nr. 837.050.17). It was designed and conducted in accordance with the recommendation of CONSORT 2010 (Supplementary Materials File S1, CONSORT 2010 checklist) and registered with an international randomized controlled trial number (NCT06508879). As a single-center prospective randomized controlled (new treatment vs. standard-of-care) parallel trial, it was conducted in the Department of Cardiovascular Surgery of the University Hospital of Mainz, Germany. Table 1 lists inclusion and exclusion criteria. These have been used to generate two homogenous groups to increase the power of this study.

All patients who were scheduled for elective CABG in our department and who met the inclusion criteria mentioned in Table 1 were informed about the study verbally after their arrival and prior to their surgery. Those who approved of taking part in the study were further educated by our team before giving their written approval at least 24 h prior to the surgery.

Random allocation of the patients to either the control or the test group was performed after obtaining the patients’ written consent. Simple randomization with neither restriction nor stratification using a computer random number generator was used to generate a 1:1 allocation ratio. The randomization was performed by the study team. Patients were blinded until the time of the intervention (application of wound dressing after the surgery). 

### 2.1. Study Groups

**Figure 1:**
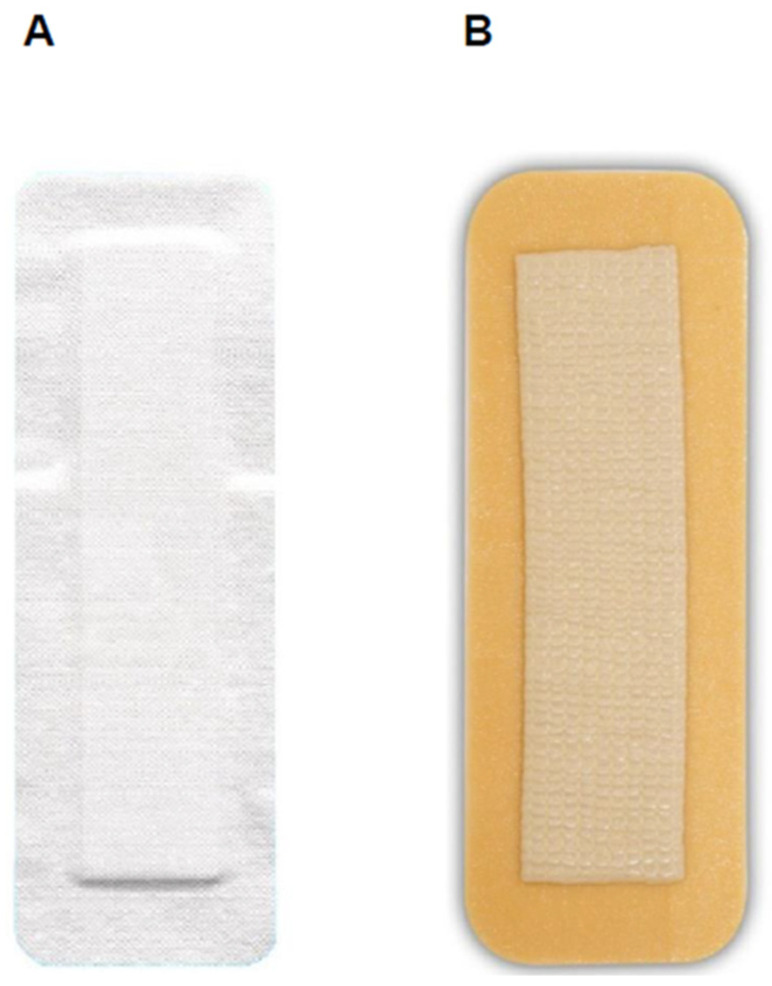
The wound dressing used in this study. (**A**) Standard-of-care wound dressing (sterile gauze and tape). (**B**) Occlusive hydrocolloid silver-containing wound dressing (Aquacel Ag Surgical^®^).

The patients were randomly allocated to one of the two groups:

Control group: a standard wound dressing composed of sterile gauze and adhesive tape was applied under sterile conditions (Figure 1A). In the absence of bleeding, excessive wound secretion, or detachment, the dressing was left on the wound until the second postoperative day (POD 2); otherwise, it was changed immediately. Starting on POD 2, the dressing was changed daily under aseptic conditions until the wound remained dry (between POD 3–5). In the absence of bleeding, excessive wound secretion, or detachment, the wound was then left uncovered. This practice corresponded to the standard treatment of cardiothoracic patients in our department at the time of study initiation.

Test group: directly after closing the wound, an occlusive hydrocolloid silver-containing wound dressing (Aquacel Ag Surgical^®^, ConvaTec, Oklahoma City, OK, USA) was applied under sterile conditions (Figure 1B) and, in the absence of bleeding, excessive wound secretion, or detachment, was maintained on the wound until POD 5, at which time the dressing was removed. In case of bleeding, excessive wound secretion, or detachment, the dressing was removed, the wound was cleaned, and a new sterile hydrocolloid wound dressing was applied under aseptic conditions.

Presurgical preparation was identical in both groups and no further intervention in patient treatment was made (for example, the same postoperative sugar control strategy was applied in both groups).

### 2.2. Surgical Technique

All patients underwent classical median sternotomy under general anesthesia (propofol, fentanyl, and sevoflurane). A protective adhesive plastic sheet was applied routinely after disinfection of the skin with povidone iodine. All patients received prophylactic antibiotics (1–2 g cefazoline or cefuroxime) directly before and after the incision, and 8 h later. Further antibiotic treatment was administered only when the medical situation necessitated it. 

One or both internal thoracic arteries were harvested using an electrical knife or ultrasonic scalpel, depending on the surgeon’s preference. Cardiopulmonary bypass (CPB) with a hollow-fiber membrane oxygenator and roller pump was used. The body temperature was kept mildly hypothermic at 34 °C or normothermic. Heparin was administered at 300 IU/kg or more to achieve an activated thrombin time (ACT) of >400 s. Sternal closure was performed with stainless steel wires. The fascia was closed with interrupted non-resorbable multifilament sutures (vicryl 0, Ethicon, bridgewater, OH, USA), the subcutaneous tissue with continuous multifilament sutures (vicryl 2-0, Ethicon), and the skin with a continuous intracutaneous suture (monocryl 4-0, Ethicon).

### 2.3. Study Outcomes

**Figure 2:**
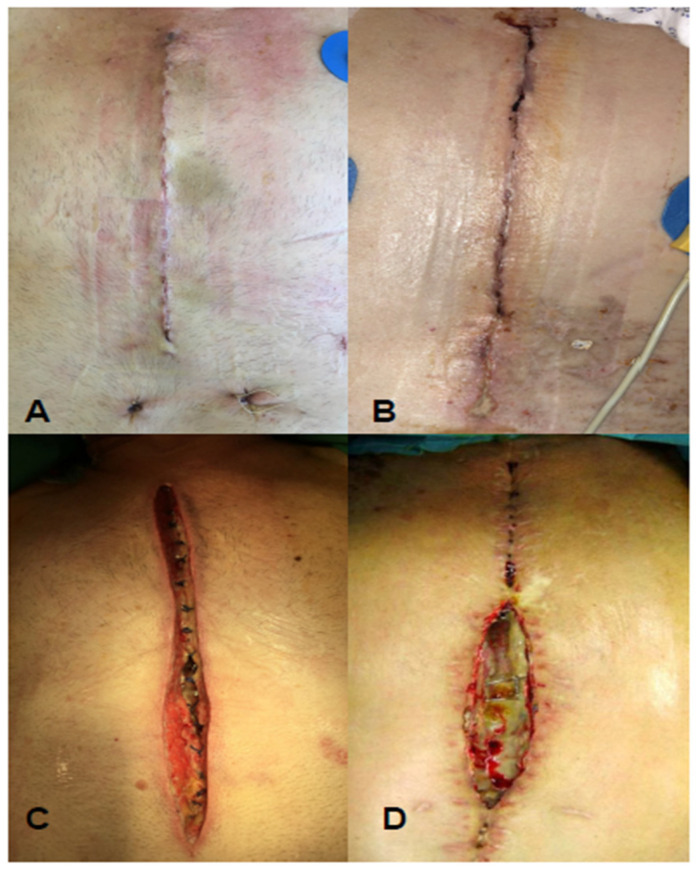
Examples of SSIs after median sternotomy. (**A**) Uncomplicated healing. (**B**) Superficial SSI with purulent discharge from the distal wound. (**C**) Deep SSI with sternal involvement (osteomyelitis). (**D**) Deep SSI with complete sternal dehiscence and mediastinitis.

Both the primary and the secondary endpoints were assessed up to the 30th postoperative day (POD 30). The primary endpoint was the overall rate of incidence of surgical site infection (SSI). Secondary endpoints were (i) superficial SSI, (ii) deep SSI, (iii) mediastinitis, (iv) surgical wound revision, or (v) sternal refixation (Figure 2). For the definition of an SSI and its classification, we followed the recommendations of the US Centers for Disease Control and Prevention (CDC) [21]. These recommendations divide SSIs into three groups—(i) superficial incisional SSIs that involve only skin and pre-sternal tissue, (ii) deep incisional SSIs that involve the sternum, and (iii) mediastinitis. The incidence of allergic reactions was also included.

### 2.4. Follow-Up and Data Storage

Patients in both groups were examined daily by the study team until the patient’s discharge. Dressings were checked for integrity, signs of bleeding, excessive wound secretion, detachment, infection, or allergy. The patients were questioned about their subjective opinion of discomfort. After discharge, all patients were contacted on the 30th postoperative day for a final evaluation. Furthermore, most patients visited our outpatient clinic for a general follow-up. Electronic entries were made and secured by the study team directly to the study database, which was maintained at the University Hospital of Mainz’s central database. 

As this was a “per protocol analysis”, patients who failed to adhere to the protocol were excluded. Table 1 lists the exclusion from analysis criteria that were applied.

### 2.5. Statistical Analysis

Statistical analyses were performed using Microsoft Excel 2016 (Microsoft, Redmond, CA, USA), XLSTAT statistical and data analysis solution (Addinsoft, New York, NY, USA), and GraphPad Prism 9.2.0 (GraphPad, San Diego, CA, USA). Categorical variables were presented by frequencies and rates. Quantitative variables, which follow the Gaussian distribution, were described by their arithmetic means and standard deviation. Non-Gaussian quantitative variables were reported by the median and 1st and 3rd quantile. Gaussian distribution was determined by the Kolmogorov–Smirnov test and by observing the histograms. The homogeneity between the groups was tested utilizing the Student’s *t*-test when the distribution was Gaussian and the Mann–Whitney test for continuous variables, utilizing Fisher’s exact test for binomial variables.

To reach a statistical power of 0.8 with an alpha value of 0.05 and after reviewing our results over the last five years (which revealed an SSI rate of almost 14%), a total of ~420 patients was required to demonstrate an effect of 0.5 in the reduction of surgical site infections. The study endpoints were tested utilizing Fisher’s exact test.

## 3. Results

**Figure 3:**
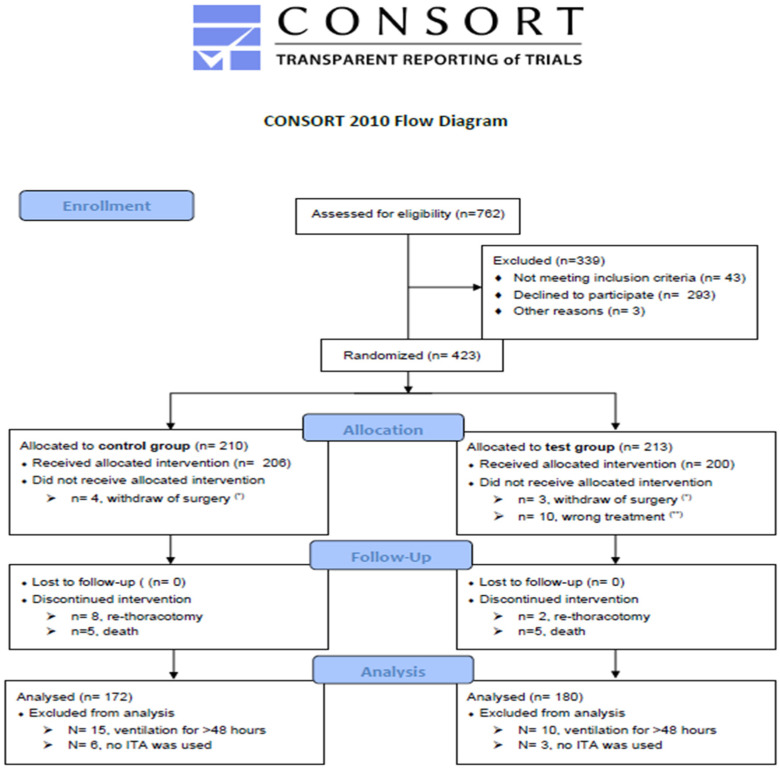
The CONSORT 2010 Flow Diagram [36]. (*) Patients who withdrew their consent and rejected the surgical treatments. Those patients end up either discharged or transferred to interventional treatment (such as PTCA). (**) Patients who were assigned to the test group but did not receive the assigned bandage due to failure of the surgical team to adhere to the study (they ended up receiving the standard treatment)—CONSORT 2010 Flow Diagram template courtesy of https://www.consort-spirit.org, accessed on September 2019.

The study began in January 2018 and ended in August 2019 after enrolling 423 patients. Figure 3 provides a structured flowchart according to the recommendations of CONSORT 2010 [36]. After applying the exclusion criteria, 352 patients were included in the final analysis.

The characteristics of the two groups (Table 2) demonstrate similar preoperative morbidities with no significant differences except for a higher rate of cerebral vascular disease in the control group (10.5 vs. 4.4%, *p* = 0.041). There were no differences in the surgical profiles between the two groups (Table 3).

There were no significant differences in the incidence of any type of surgical site infection between the two groups (Table 4).

In the test group, a wound dressing change in the first 5 days was necessary in 17 patients (see Table 4 for more details). Only one of these patients (who was in the test group and required a dressing change) developed a surgical site infection (deep form which required sternal refixation). In the remaining 163 patients (91%), no dressing change was required.

## 4. Discussion

Surgical site infections (SSIs) are the second most common and costly healthcare-associated infection (HAI) in European hospitals [37]. They lead to 2–11 times higher mortality risk [38], a significant increase in postoperative hospital days [39], and therefore to 1.4–3 times higher care costs compared to surgical patients without postoperative wound healing disorders [40,41].

Up to 60% of SSIs are preventable by using evidence-based guidelines [42,43]. All current guidelines make the few following evidence-based recommendations as the most important strategies for preventing wound healing disorders:

1. decolonization with intranasal antistaphylococcal agents and antistaphylococcal skin antiseptics for high-risk procedures [44,45,46],
2. hair removal outside of the operating room using clippers [47,48],
3. use of chlorhexidine gluconate and alcohol-based skin preparation [49,50], and
4. perioperative glycemic control <150 mg/dL [51,52].

As our clinic already follows the general evidence-based guideline recommendations for the prevention of SSIs that are applicable in the field of cardiac surgery, we have focused our particular attention on postoperative wound care in order to further reduce our incidence of postoperative wound healing disorders.

While the KRINKO recommendations from 2018 attribute the main focus for the occurrence of SSIs to inoculation during surgery [53,54,55], the WHO guidelines from 2018 and even more recent publications identify postoperative contamination as a relevant factor for the occurrence of SSIs [56,57]. Various types of dressings are available to avoid this postoperative contamination. Therefore, the dressing should act as a physical barrier to protect the wound from external contamination until the wound becomes impermeable to microorganisms after three to six days and it should also serve to absorb exudate from the wound and help to keep it dry [19]. In 2013, an update of the UK-based NICE guidelines suggested that silver nylon dressings might be more effective than gauze [23]. For these reasons, we opted for an occlusive hydrocolloid silver-containing wound dressing (OHSCWD) in our study, as we believed that its properties offered the highest postoperative contamination reduction, absorption, and antimicrobial function. In addition, as already reported in other studies, we have noted that occlusive hydrocolloid dressings generally reduce the number of dressing changes considerably. This is usually very positively received by the patients, who feel that the occlusive hydrocolloid dressing is more pleasant and comfortable [58,59]. In addition, the OHSCWD requires less time and effort for medical staff, which is becoming an increasingly important factor in the current climate [60]. 

The costs for both kinds of dressings also level out with increased wound secretion from six changes of the standard dressing (EUR 20 vs. EUR 3.50).

Currently, as only a very few large, high-quality trials are investigating different types of dressings with SSI prevention as a primary outcome, there are still no specific recommendations or guidelines regarding the type of surgical dressing [19]—especially in cardiac surgery [61]. Therefore, it was emphasized in the WHO Guidelines in 2018 that further research to confirm the effectiveness of modern types of dressings is urgently required and there is a special interest in investigating the use of silver-containing dressings in cardiac surgery with regard to SSI prevention [19].

Our study has shown that an occlusive hydrocolloidal silver-containing wound dressing (OHSCWD) is equivalent to standard sterile gauze and tape dressing in patients undergoing median sternotomy for cardiac surgery. 

No differences were found between the two groups in terms of demographics, cardiovascular risk factors, intraoperative procedures, and postoperative care. In particular, with regard to all endogenous (e.g., age, obesity, and diabetes) and exogenous risk factors for the development of SSIs and the recommendations for their prevention (e.g., decolonization, type of preoperative hair removal, skin preparation, and perioperative glycemic control) reported in the current guidelines, all patients were treated according to the same internal hospital standard [19,20,21,22].

Nevertheless, our study has some limitations.

(I) The patients were blinded until the time of the intervention (application of the bandage after surgery). Due to the nature of this study, it was not possible to conceal the treatment after the procedure, as patients could see their bandages and find out their allocation. Using methods to conceal the dressings (covering with neutral dressing) would have changed the nature of the dressings and affected the treatment. The study team could also see the bandages and was therefore not blinded.
(II) In addition, overestimation of the effect of the dressing on reducing the incidence of SSIs during the design of the study may have resulted in the number of subjects required for this study (n~420) being miscalculated. The number of patients included in the final analysis was even lower (n = 352) due to the predefined exclusion criteria and the “analysis per protocol” design. This may have unintentionally caused our study to be underpowered.
(III) This study was our first experience with the new dressing, which may have led to delays in detecting signs of wound infection. Cost-effective standard sterile gauze and tape are permeable to fluids and gases and retain only small amounts of fluid, allowing early detection of incipient wound secretion and more frequent dressing changes. Aquacel Ag Surgical^®^ has a hydrocolloid adhesive layer and a polyurethane cover. Due to its good occlusion, this absorbent hydrofiber layer can form a moist chamber in the event of increased wound secretion. In the case of consecutive fluid overload, we would recommend a more aggressive approach to changing the hydrocolloid dressing, namely at the first signs of fluid overload, based on our experience with this study.

Although, more frequent dressing changes, if not performed with the necessary care, may favor contamination of the surgical wound [62] and thus an occlusive dressing that is completely sealed from the atmosphere and does not need to be changed daily has been advocated in several studies [26,60,63], another reason for overestimating the effect of the dressing and thus possibly explaining our results could have been the location of the chest drainage tubes close to the caudal wound pole. This could have impaired the occlusive properties of the dressing and favored the penetration of bacteria at this point. In our experience, most SSIs start at the caudal wound area. We also noted a tendency for over-watering and spontaneous detachment of the hydrocolloid dressing during the summer season, which may also weaken its protective properties.

The incorporation of silver ions into the absorbent hydrofiber layer (sodium carboxymethyl cellulose with 1.2% silver ions) of the OHSCWD should reduce microbacterial colonization [30]. The ability of its ions to interact with various proteins and cause denaturation explains this effect [64]. Silver, used since ancient times, has antibacterial properties whereas eukaryotic cells are less susceptible to silver toxicity than prokaryotes and thus require an increased silver concentration for a toxic effect. Studies on wound dressings containing silver preparations have demonstrated that the majority of the Ag+ ions released into the wound are not available for systemic absorption and thus cause no significant toxicity or permanent organ damage. Other studies that point to the genotoxicity or mutagenicity of silver have been proven negative or at high concentrations only [29]. Because the counter-ions to the silver are the dressing fibers in this dressing, the problems of toxicity are avoided [31]. 

In summary, it must be said that the contribution of postoperative wound treatment to the development of SSI is probably significantly lower than estimated. The above-mentioned pre- and intraoperative factors (decolonization, type of preoperative hair removal, skin disinfection, and perioperative blood glucose level) seem to play a much greater role.

## 5. Conclusions

In general, the contribution of postoperative wound care to the development of SSIs must be considered. In the overall context of all pre-, intra-, and postoperative factors, this appears to play a subordinate role for both patients and institutions. Nevertheless, applying an occlusive hydrocolloidal silver-containing wound dressing after median sternotomy in cardiac surgery patients offers equivalent wound protection to standard care (sterile gauze and adhesive tape). By reducing the number of dressing changes, occlusive hydrocolloidal silver-containing dressings could be a good alternative to standard dressings from the point of view of the patient, the staff, and the clinic.

## Figures and Tables

**Table 1:** Inclusion and exclusion criteria.

Criteria for Inclusion in Randomization	Criteria for Exclusion from Analysis
Coronary artery bypass surgery Elective and primary surgery (no reoperations) Complete median sternotomy Age under 90 years Weight in the range of 50–140 kg No known allergy to silver or other wound dressings No immunosuppressive therapy or hormonal substitution therapy, except for thyroid hormone Capacity to consent	Failure of medical personnel to adhere to the study protocol, e.g., not applying the right wound dressing according to the study protocol Patient’s own decision ^1^ Lack of harvesting of at least one internal thoracic artery Peri- and postoperative ventilation for >48 h Re-thoracotomy for reasons other than SSI, e.g., bleeding Mortality within the first 30 days

^1^ Some patients withdraw their consent for various reasons and canceled their surgery after their enrollment in this study.

**Table 2:** Patient characteristics.

	Control G	Test G	*p*-Value
	[N = 172]	[N = 180]	
Number of males (%)	140 (81.4)	142 (79.8)	0.593
Mean age ± SD	68.5 ± 9.9	67.3 ± 9.1	0.241
Mean BMI ± SD	28.1 ± 4.9	28.4 ± 4.6	0.610
Diabetes mellitus (%)	64 (37.2)	75 (41.7)	0.383
IDDM (%)	37 (21.5)	53 (29.4)	0.087
Chronic pulmonary disease (%)	20 (11.6)	15 (8.3)	0.374
Nicotine abuse (%)	44 (25.6)	42 (23.3)	0.710
Arterial hypertension (%)	144 (83.7)	139 (77.2)	0.221
Hyperlipidemia	105 (61.0)	106 (58.9)	1.000
treated with statin	29 (16.9)	41 (22.8)	0.181
Peripheral artery disease (%)	24 (14.0)	22 (12.2)	0.752
Cerebral vascular disease (%)	18 (10.5)	8 (4.4)	0.041
Estimated creatinine clearance	85.3 ± 29.5	81.9 ± 30.0	0.289
LVEF category			
Reduced (30–49%)	31 (18.0)	35 (19.4)	0.785
Poor (<30%)	15 (8.7)	13 (7.2)	0.696
NYHA			
III (%)	46 (26.7)	45 (25.0)	0.808
IV (%)	6 (3.5)	2 (1.1)	0.168
Median of Euroscore II [IQR]	1.20% [0.84–2.05]	1.24% [0.80–1.97]	0.821

BMI: body mass index, SD: standard deviation, IDDM: insulin-dependent diabetes mellitus, LVEF: left ventricular ejection fraction, NYHA: New York Heart Association classification, IQR: interquartile range.

**Table 3:** Surgical parameters.

	Control G	Test G	*p*-Value
	[N = 172]	[N = 180]	
Combined surgery	33 (19.2)	29 (16.1)	0.488
Off-pump surgery	2 (1.2)	4 (2.2)	0.685
Count of ITA			
sITA	43 (25.0)	42 (23.3)	0.804
dITA	129 (75.0)	136 (75.6)	0.804
Harvesting technique			
classic	31 (18.0)	26 (14.4)	0.469
skeletonized (with ultrasound knife)	141 (82.0)	152 (84.4)	0.469
Number of anastomoses	2.8 ± 0.7	2.7 ± 0.8	0.091
Median of skin-to-skin time [IQR]	228 [196–274]	226 [191–280]	0.929
Median of ventilation time [IQR]	9 [7–13]	9 [6–12]	0.714

Combined surgery: coronary artery bypass surgery combined with further procedures such as aortic or mitral valve replacement, ITA: internal thoracic artery, sITA: single ITA, dITA: double ITA, IQR: interquartile range.

**Table 4:** Group comparisons.

	Control G	Test G	*p*-Value
	[N = 172]	[N = 180]	
All SSIs	15 (8.7)	19 (10.6)	0.591
superficial SSI	9 (5.2)	12 (6.7)	0.655
deep SSI	6 (3.5)	7 (3.9)	1.000
mediastinitis	3 (1.7)	1 (0.6)	0.364
Surgical revision	11 (6.4)	14 (7.8)	0.680
Sternal refixation	6 (3.5)	7 (3.9)	1.000
Wound dressing change *	172 (100.0)	17 (9.4)	<0.0001
excessive wound secretion	-	5 (2.8)	-
spontaneous detachment	-	4 (2.2)	-
delirium	-	2 (1.1)	-
suspicion of allergy	-	1 (0.6)	-
application failure	-	1 (0.6)	-
CPR	-	1 (0.6)	-
of them, SSI **	-	1 (0.6)	-

SSI: surgical site infection. CPR: cardiopulmonary resuscitation. (*) Patients in the test group whose dressing had to be changed in the first five postoperative days due to the different reasons listed above. (**) Patients in the test group who developed SSI after changing their dressing in the first five postoperative days.

## Data Availability

Data are available upon request from the corresponding author.

## Supplementary Materials

The following supporting information can be downloaded at: https://www.mdpi.com/article/10.3390/life14091061/s1, File S1: The CONSORT 2010 checklist of information to include when reporting a randomized trial can be downloaded.
